# Retraction Notice

**DOI:** 10.14252/foodsafetyfscj.D-24-00004

**Published:** 2024-03-22

**Authors:** Yasushi Yamazoe

**Affiliations:** Editor-in-Chief, *Food Safety*

## RETRACTED

RETRACTED: Development and Evaluation of Fluorescence Immunochomatography for Rapid and Sensitive Detection of Thermophilic *Campylobacter*. *Food Safety*. 2021; 9(3): 81–87. doi: 10.14252/foodsafetyfscj.D-21-00006.

Hiroshi Asakura, Junko Sakata, Yoshimasa Sasaki, Kentaro Kawatsu

Food Safety decided to retract this article in which the primary author misconducted as reported from the primary author’s affiliation.

March 22, 2024

